# 25 Years of Digital Health Toward Universal Health Coverage in Low- and Middle-Income Countries: Rapid Systematic Review

**DOI:** 10.2196/59042

**Published:** 2025-05-29

**Authors:** Bry Sylla, Ouedraogo Ismaila, Gayo Diallo

**Affiliations:** 1 Public Health Department Nazi Boni University Bobo Dioulasso Burkina Faso; 2 Bordeaux Population Health Inserm 1219 University of Bordeaux Bordeaux France

**Keywords:** digital health interventions, low- and middle-income countries, universal health coverage, medical informatics, health information systems

## Abstract

**Background:**

Over the last 25 years, digital health interventions in low- and middle-income countries have undergone substantial transformations propelled by technological advancements, increased internet accessibility, and a deeper appreciation of the benefits of digital tools in enhancing health care availability.

**Objective:**

This study aims to examine the evolution, impact, and prospects of digital health interventions in low- and middle-income countries, highlighting their role in improving health care accessibility and equity.

**Methods:**

A retrospective analysis of digital health initiatives scanning the past two and a half decades focused on the progression from basic SMS platforms to sophisticated mobile health apps and other health digital interventions. Relevant literature and case studies were reviewed to elucidate key milestones, successes, challenges, and opportunities in advancing digital health initiatives in low- and middle-income regions.

**Results:**

Digital health initiatives in low- and middle-income countries initially targeted specific health concerns, such as malaria diagnosis and treatment, through text-based platforms, demonstrating their efficacy in reaching remote and marginalized communities. With the proliferation of mobile phone ownership and internet access, these interventions evolved into comprehensive mobile health apps, facilitating self-care support, patient education, chronic disease monitoring, and remote consultations. The COVID-19 pandemic further accelerated the adoption of digital health interventions, particularly in disseminating health information, supporting contact tracing efforts, and enabling virtual consultations to alleviate strain on health care systems.

**Conclusions:**

The future of digital health interventions in low- and middle-income countries holds immense promise, fueled by emerging technologies such as artificial intelligence, machine learning, and blockchain. However, challenges persist in ensuring equitable access to digital health technologies, addressing disparities in digital literacy, and establishing robust health care infrastructure. Collaboration among governments, health care providers, technology innovators, and communities is essential to overcome these challenges and harness the full potential of digital health to improve health care outcomes in low- and middle-income countries.

## Introduction

### Background

Digital health initiatives hold significant potential for transforming health care delivery in low- and middle-income countries (LMICs). Over the past two decades, the literature has documented how these initiatives can enhance health systems by improving early warning systems for disease outbreaks, strengthening emergency preparedness, and expanding access to essential health care services [1]. Moreover, digital solutions, ranging from basic SMS-based platforms to more advanced mobile health (mHealth) apps, have been used to address pressing health care challenges, particularly in remote and underserved communities [2].

A large and growing body of research highlights the potential of digital health interventions to address specific health challenges such as infectious disease surveillance, maternal and child health, and chronic disease management. In addition, digital health has played a key role during worldwide health crises such as the COVID-19 pandemic, where it has enabled virtual consultations and supported efforts to trace contacts and disseminate accurate health information. However, these achievements are often contextual, responding to isolated health problems, while the broader goal of universal health coverage (UHC) remains elusive in many LMICs [3].

Despite these advances, there remains a considerable gap in understanding how digital health initiatives specifically contribute to realizing UHC in LMICs. While numerous studies highlight the effectiveness of digital tools in specific interventions such as malaria treatment or chronic disease management, there is less clarity on how these technologies integrate into broader health systems to support UHC goals. In addition, disparities in digital literacy, infrastructure, and equitable access to technology continue to present significant challenges, further complicating the pathway toward achieving UHC.

This paper seeks to address this gap by examining the role of digital health initiatives in advancing UHC in LMICs. Through a review of key case studies and an analysis of the evolution of digital health interventions over the past two decades, this study will explore both the opportunities and challenges that arise with the implementation of these initiatives [4]. Special attention will be given to understanding how digital health can bridge gaps in health care delivery, particularly in marginalized and remote communities, and what strategies are required to ensure the long-term success and integration of these technologies into existing health systems.

By doing so, the aim is to contribute to the growing body of literature on the potential of digital health to transform health care systems and support UHC objectives in these contexts.

### Digital Health Initiatives

Digital health initiatives encompass a diverse array of technologies and strategies aimed at leveraging digital tools to enhance health and health care delivery, focusing on improving effectiveness, efficiency, accessibility, safety, and personalization [5]. At its core, digital health involves the integration of information and communication technologies into various health care domains, including diagnosis, treatment, monitoring, and management.

This concept is grounded in the belief that technological advancements, such as mobile devices, wearable sensors, artificial intelligence (AI), and data analytics [6], have the potential to transform traditional health care practices and systems [7]. These technologies, including electronic health records (EHRs), virtual visits, mHealth, wearable technology, digital therapeutics, AI, and machine learning, empower health care providers to streamline processes, increase efficiency, and deliver more personalized and effective care to patients [8]. Moreover, clinical decision support systems are integral to the modern digital health ecosystem. These systems offer tools that enhance clinical decision-making through evidence-based recommendations, predictive analytics, and real-time data integration. When integrated with interoperable health IT frameworks, clinical decision support systems can significantly improve patient outcomes by enabling seamless data exchange between different health care providers and systems. This interoperability ensures that vital information is easily accessible, thus improving care coordination and reducing the likelihood of error [9]. A pivotal component of digital health is the digitization of health records and information systems, exemplified by the widespread adoption of EHRs, facilitating quick and secure access to patient data, thereby improving care coordination and clinical decision-making [10]. The concept of “Loose Coupling” in EHR systems, as exemplified by the Ayushman Bharat Digital Mission approach in India, offers a flexible alternative to monolithic EHR implementations. This strategy enables the integration of diverse health IT systems without necessitating a single, centralized platform, thus allowing for scalable and adaptable health care solutions across varied health care settings [11]. Furthermore, digital health encompasses remote monitoring and telemedicine solutions [12], which are particularly valuable for reaching underserved populations and reducing barriers to care [13]. mHealth apps and devices play a significant role in empowering individuals to proactively manage their health, facilitating tracking of health metrics, adherence to treatment plans, and informed lifestyle decisions. These solutions are accessible and scalable, promoting behavior change, improving health outcomes, and reducing health care costs [14]. AI and machine learning are increasingly integrated into digital health solutions, enabling predictive analytics, disease detection, and personalized treatment recommendations [15]. These technologies can potentially revolutionize clinical decision support, drug discovery, and precision medicine approaches. In LMICs, community health workers are crucial in bridging the digital divide, particularly in remote and underserved areas. By using digital health tools, community health workers can provide essential health services, monitor patient progress, and facilitate communication between patients and formal health care providers, thereby enhancing health care delivery in these regions [16].

Digital health has the immense potential to revolutionize health care by leveraging technology to improve access, efficiency, and quality of care. By embracing digital solutions, health care systems can address challenges and transition toward a patient-centered, data-driven, and sustainable health care delivery model.

### Importance of Advancing Digital Health in LMICs

Health care disparities in LMICs persist as a significant challenge, characterized by unequal access to medical services, limited infrastructure, and inadequate resources [17], [18]. These disparities exacerbate the already-existing health burdens, contributing to higher morbidity and mortality rates [19]. However, the advent of digital health technologies presents a promising solution to bridge these gaps and improve health care outcomes in LMICs.

The distribution of mobile phones and related technology is rapidly growing in LMICs. For example, India and sub-Saharan Africa will account for around half of new mobile subscribers globally over the 2022-2030 period [20], offering numerous opportunities for digital health to supply value to individuals and communities. However, despite these opportunities, digital health initiatives often fail to reach vulnerable populations due to structural, practical, commercial, and economic barriers [21].

Limited resources, infrastructure, information, and knowledge, as well as embedded structural complexities such as racism and power imbalances, contribute to inequitable access to digital health services. In addition, digital health applications may not be well suited to individuals with limited basic literacy skills such as reading and writing, further exacerbating disparities [22].

Despite their greater need for support, individuals in vulnerable situations often benefit less from digital health services. This exclusionary trend has become more apparent during the COVID-19 pandemic, widening the digital divide and exacerbating existing health care inequalities across continents, countries, and regions [23]. Recognizing the importance of reducing global health inequities, numerous global initiatives and national programs are committed to making digital health accessible to all citizens and communities. However, these initiatives often lack concrete examples of inclusive digital health projects on the ground and struggle to connect to create synergies.

To address these challenges, it is crucial to facilitate mutual knowledge exchange, both globally and locally, and to link initiatives to apply academic knowledge into practice. Collaboration between academia, the private sector, nongovernmental organizations, and citizens is essential to achieve the goal of “Digital Health for All” [24]. This collaboration should involve bundling knowledge from a wide range of disciplines, implementing this knowledge in concrete projects in collaboration with communities and health care providers, and using existing frameworks to ensure health equity.

Furthermore, to properly evaluate and facilitate the exchange of knowledge and its implementation, it is essential to develop a joint data infrastructure [24]. By combining data from various domains, such as health care, education, work, and income, it is possible to map out inequality and investigate how connecting data can promote well-being and structural change within vulnerable groups.

Digital health innovations have the potential to overcome geographical and practical barriers to health care, supporting UHC. However, to ensure accessibility and affordability of digital health for all, policy makers, health care professionals, and patients must collaborate, develop, implement, and share digital health initiatives that address the health needs of vulnerable populations [24]. Through inclusive approaches and concerted efforts, we can bridge health care disparities in LMICs and improve health outcomes for all citizens.

### Statement of Problem and Purpose of Article

The rapid rise of digital health initiatives in LMICs over the last two and a half decades offers a great opportunity to improve access to health care and equity. Still, there is an urgent need for retrospective analysis to effectively shape future strategies. This study examines the evolution, impact, and prospects of digital health interventions in these regions, highlighting their role in promoting UHC. Despite their potential, challenges such as limited resources, infrastructure, and digital literacy hinder equal access to digital health services and exacerbate existing inequalities. In examining specific case studies and successes, this analysis aims to provide insights into the transformative power of digital health initiatives and address the barriers to their widespread adoption. In working together across stakeholders, including governments, health care providers, technology innovators, and communities, we can overcome these challenges and realize the full potential of digital health to improve health care and promote the well-being of vulnerable populations.

## Methods

### Overview

This study is a rapid systematic literature review on digital health interventions targeting UHC in LMICs [25]. We employed the PICO (Population, Intervention, Comparison, and Outcome) framework to structure our research question [26], focusing on digital health (Population), health interventions (Intervention), and impact on UHC (Outcome; Comparison is not applicable). Our research question addresses the impact of implementing digital health interventions on health system improvement in LMICs.

### Research Rabbit AI Mapping Tool for Papers

We used Research Rabbit, a citation-based literature mapping tool, to conduct the literature review [27]. Research Rabbit includes Semantic Scholar and PubMed, with over 200 million papers from various scientific fields [28]. For our search strategy, predefined keywords were used to identify relevant literature, including “digital health,” “health information systems,” “mobile health,” “universal health coverage,” “developing countries,” and “low- and middle-income countries.”

### Paper Inclusion and Exclusion

We conducted a rapid systematic review following the PRISMA (Preferred Reporting Items for Systematic Reviews and Meta-Analyses) guidelines to determine the inclusion and exclusion of papers. Using the PRISMA framework, we meticulously screened abstracts and titles of papers based on specific criteria outlined in Textbox 1. The review process was conducted by two independent researchers at each stage, ensuring the robustness and reliability of the findings.

Inclusion criteria were defined as papers published in either English or French, focusing on digital-based health solutions contributing to UHC, and implemented in LMICs (excluding China and India) according to the World Bank classification. China and India were excluded from this study due to their unique and advanced digital health ecosystems, which significantly differ from those in other LMICs. Both countries have implemented large-scale national programs, such as China’s national health information system and India’s Ayushman Bharat Digital Mission, which feature comprehensive EHR systems and integrated telemedicine platforms. These initiatives, supported by substantial government investment and robust digital infrastructure, place China and India in a distinct category of digital health readiness. Including them in the analysis could skew the comparative results, as their challenges and outcomes in digital health are not directly comparable to those in other LMICs. We considered papers published between 1999 and 2024, aligning with PRISMA’s emphasis on comprehensive search strategies and transparent reporting practices*.*

**Criteria for inclusion and exclusion of papers.**

**Inclusion Criteria**

- Articles published in English and French
- Articles focusing on low- and middle-income countries, excluding China and India
- Articles addressing the impact on universal health coverage
- Articles published between 1999 and 2024

**Exclusion Criteria**

- Papers published in other languages
- Papers focusing on high-income countries
- Papers not focusing on improving universal health coverage
- Papers published outside the specified period

### Data Extraction

Data extraction was performed based on predefined variables, including author and publication year, country or region of implementation, technology characteristics, sector of intervention, and the paper’s contributions to UHC. The study aligns with the World Health Organization’s definition of UHC, which includes ensuring that everyone receives essential health services without experiencing financial hardship. This entails investing in health service delivery systems, the health workforce, facilities, communication networks, health technologies, information systems, quality assurance mechanisms, and governance and legislation [29].

### Analysis and Interpretation of Results

Quantitative analysis of extracted data was conducted using SPSS 28 (IBM Corporation) software. Absolute and relative frequencies were computed to analyze the data quantitatively, providing insights into the prevalence and distribution of various factors related to digital health interventions and their impact on UHC. The results, including quantitative summaries, are presented through tables and figures. These analyses contribute to a comprehensive understanding of the effectiveness and implications of digital health interventions in promoting UHC in LMICs.

## Results

### Included Studies

**Figure 1:**
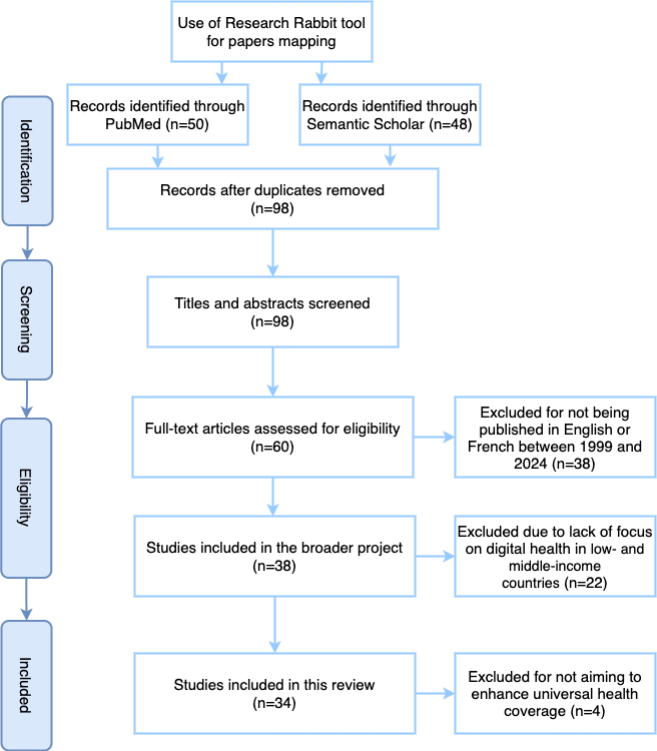
PRISMA (Preferred Reporting Items for Systematic Reviews and Meta-Analyses) flowchart of study selection for a scoping review.

Using Research Rabbit, a citation-based literature mapping tool, we conducted an extensive search. As illustrated in Figure 1, during the identification phase, databases and tools, including the Research Rabbit tool, were used to map documents. A total of 50 records via PubMed and 48 records via Semantic Scholar were identified. After removing duplicates, there were 98 unique records. In the screening phase, the titles and abstracts of the 98 records were screened. Of these, 38 were excluded because they had not been published in English or French between 1999 and 2024. In the eligibility phase, the full-text articles (60 in total) were checked for suitability. Of these, 22 were excluded because they did not focus on digital health in LMICs. This left 38 studies to be included in the wider project. In addition, 4 studies were excluded because they did not focus on improving UHC, leaving 34 studies (Table 1) to be included in this review.

**Table 1:** Included digital health initiatives and the characteristics of technology used.

Authors and year	Implementation countries	Technology used
Azubuike and Ehiri, 1999 [30]	Low- and middle-income countries (unspecified)	HIS^a^
Braa et al, 2004 [31]	Mongolia, Nigeria, Tanzania (and Zanzibar), Botswana, Cuba, Malawi, Vietnam, South Africa, Mozambique, and Ethiopia.	HIS
Kimaro and Nhampossa, 2004 [32]	Mozambique and Tanzania	HIS
Simba and Mwangu, 2004 [33]	South Africa, Mozambique, Malawi, and Cuba	ICT^b^
Braa et al, 2007 [34]	Mongolia, Tanzania (and Zanzibar), Botswana, Cuba, Malawi, Vietnam, South Africa, and Ethiopia	HIS
Krickeberg, 2007 [35]	Vietnam	HIS
Weibel et al, 2008 [36]	Chad	mHealth^c^ surveillance with biometrics
Nicholas, 2008 [37]	Nigeria	Mobile technology devices
Asangansi and Braa, 2010 [38]	Nigeria	Open-source, Java-based mobile platform for health information
Blaya et al, 2010 [39]	Kenya, Peru, Iran, Malawi, Haiti, South Africa, Nicaragua, Cambodia, Tanzania, and Malaysia	eHealth uses EHRs^d^, mobile devices, and clinical decision support
Nguyen and Nyella, 2010 [40]	Tanzania and Vietnam	DHIS^e^, OpenEPR, and electronic medical records repository
Moodley et al, 2011 [41]	South Africa, Mozambique, and Rwanda	Health Enterprise Architecture Framework for African countries’ information systems
Soar et al, 2012 [42]	Fiji	Patient Administration System for Patient Management
Mudaly et al, 2013 [43]	Tanzania, Rwanda, Kenya, Ghana, and South Africa	HIS for efficient patient management
Hall et al, 2014 [44]	Thailand, South Africa, Kenya, Peru, Tanzania, Uganda, Botswana, Egypt, Rwanda, Senegal, Cambodia, Bangladesh, Nigeria, Brazil, Swaziland, Pakistan, and Malawi	Mobile phones, mHealth apps, and text messages
O’Connor et al, 2015 [45]	Malawi	Android-based smartphone technology
Ishijima et al, 2015 [46]	Tanzania	HRIS^f^ and TIIS^g^
Gebre-Mariam and Fruijtier, 2018 [47]	Ethiopia	Enterprise architecture and IT governance
Eze et al, 2018 [48]	Pakistan, Kenya, Swaziland Uganda, Tanzania, Zambia, Bangladesh, Algeria, Sri Lanka Haiti, Honduras, Mexico, Iraq Argentina, Guatemala, Peru, Cameroon, Mozambique, Nepal, Brazil, Madagascar, Colombia, the Philippines, Zimbabwe, Indonesia, Seychelles, Democratic Republic of Congo, Cambodia, and Burkina Faso	mHealth solutions
Mbelwa et al, 2019 [49]	Tanzania	mHealth apps
Gebre-Mariam and Bygstad, 2019 [50]	Ethiopia	HMISh
Muinga et al, 2020 [51]	Kenya	EHR systems, data coding standards, and interoperability solutions.
Walsham, 2019 [52]	South Africa, Bolivia, and Sri Lanka	HIS and DHIS2
Hoque et al, 2020 [53]	Kenya and Sri Lanka	mHealth apps
McCool et al, 2020 [54]	Samoa, Kenya, New Zealand, and Tanzania	SMS messaging, mobile apps, virtual reality, algorithms, data management systems, job aid apps, communication platforms,
Kipruto et al, 2022 [55]	Sub-Saharan Africa	Mobile technology, telemedicine collaborations, wearables and sensors, big data, AI^i^, Internet of Things, advanced computing, and machine learning
Al-Kahtani et al, 2022 [56]	Saudi Arabia	Health information technology, EHRs, interoperability, predictive analytics, and telemedicine
Kilua, et al, 2022 [57]	Tanzania	USSD^j^ platform
Hui et al, 2022 [58]	Bangladesh, Indonesia, Malaysia, and Pakistan	Pakistan: service mapping, pneumonia app, Roman Urdu SMS chatbot, mobile game, and remote health system Malaysia: asthma self-management and clinical guideline app Indonesia: centralized FHIR^k^-based health care system Bangladesh: online questionnaire for physical activities or pulmonary rehabilitation exploration
Sumarsono et al, 2023 [59]	Indonesia	mHealth
Nguyen, 2023 [60]	Vietnam	DHIS2, Java, Microsoft Excel, web-based interfaces, data sharing standards, and interoperability
Thomas et al, 2023 [61]	Botswana and Rwanda	Vinyasa tool for mHealth solutions
De and Pradhan, 2023 [62]	Bangladesh, Nigeria, Afghanistan, Congo, Madagascar, Malawi, Pakistan, Timor-Leste, and Uganda	Mobile technology
Alfian et al, 2023 [63]	Indonesia	mHealth apps, mobile phone calls, video calls, text messages, and telemonitoring

^a^HIS: health information systems.

^b^ICT: information and communication technology.

^c^mHealth: mobile health.

^d^EHR: electronic health record.

^e^DHIS: Digital Health Information Systems.

^f^HRIS: human resource information.

^g^TIIS: training institution information systems.

^h^HMIS: health management information systems.

^i^AI: artificial intelligence.

^j^USSD: Unstructured Supplementary Service Data

^k^FHIR: Fast Healthcare Interoperability Resources.

#### User Statistics

As shown in Table 1, between 1999 and 2010, a total of 12 papers were identified that primarily addressed health care challenges in LMICs, accounting for 35.29% (12/34) of the total studies. The predominant technology emphasized in these papers was health information systems. From 2010 to 2020, a total of 28 papers were identified, accounting for 82.35% (28/34) of the included studies. These papers show a greater geographical reach and technological diversification compared to the earlier period. Digital Health Information Systems and mHealth apps emerged as important technologies during this period. In the post-2020 era, 18 papers were identified, accounting for 52.94% (18/34) of the total studies. These papers reflect an increased focus on advanced mobile technologies, telemedicine collaborations, and the integration of AI.

#### Analysis

A comprehensive review of 34 selected papers (refer to Table 2) was conducted to assess the distribution of content across 8 key categories. This review focused on both the absolute and relative frequencies of the papers within these categories. In the domain of health service delivery systems, particular attention was given to sustainable and adaptive health systems, each of which was represented by 2 papers [31,34], constituting 5.88% (2/34) of the total corpus. In the area of the health workforce, a notable planning initiative was highlighted in 1 paper [46], accounting for 2.94% (1/34) of the total papers reviewed.

**Table 2:** Overview of digital health initiatives and their contribution to universal health coverage (UHC).

Authors and year	Sector of intervention	Contribution to UHC
Azubuike and Ehiri, 1999 [30]	HIS^a^	Improve health care data
Braa et al, 2004 [31]	Action networks	Sustainable health care systems
Kimaro and Nhampossa, 2004 [32]	Data quality	Strengthen health information
Simba and Mwangu, 2004 [33]	HIS	Enhance health care access and effectiveness
Braa et al, 2007 [34]	Information infrastructures	Adaptable health care systems
Krickeberg, 2007 [35]	HIS	Integrate clinical practice
Weibel et al, 2008 [36]	mHealth^b^ surveillance	Monitor hard-to-reach populations
Nicholas, 2008 [37]	Mobile education	Enhance health care access
Asangansi and Braa, 2010 [38]	Health information management	Scale national health systems
Blaya et al, 2010 [39]	eHealth technologies	Enhance data collection
Nguyen and Nyella, 2010 [40]	Health care data management	Improve health care systems
Moodley et al, 2011 [41]	Open health architectures	Develop national information systems
Soar et al, 2012 [42]	Health information and communication technology in Fiji	Sustain health care information
Mudaly et al, 2013 [43]	Architectural frameworks	Streamline health information
Hall et al, 2014 [44]	HIS	Enhance health care access, treatment adherence, and support health workers
O’Connor et al, 2015 [45]	Supporting the LIFE Project	Enhance health care efficiency
Ishijima et al, 2015 [46]	HRH^c^ information systems	Effective workforce planning
Gebre-Mariam and Fruijtier, 2017 [47]	Enterprise architecture	Drive HIS technological changes
Eze et al, 2018 [48]	mHealth solutions	Improve health care access
Mbelwa et al, 2019 [49]	HIS	Improve the quality, accessibility, and efficiency of health care services
Gebre-Mariam and Bygstad, 2019 [50]	HMIS^d^ digitalization	Impact of HMIS digitalization
Muinga et al, 2020 [51]	mHealth interventions	Facilitate self-care management
Walsham, 2019 [52]	HIS	Emphasize data quality
Hoque et al, 2020 [53]	mHealth interventions	Improve evidence-based reporting
McCool et al, 2020 [54]	Digital health sustainability	Identify factors for integration
Kipruto et al, 2022 [55]	Digital health interventions in sub-Saharan Africa	Strengthen health systems
Al-Kahtani et al, 2022 [56]	Digital health transformation	Assess readiness for health care
Kilua, et al, 2022 [57]	Mobile vaccine registry	Improve immunization data
Hui et al, 2022 [58]	Digital health solutions	Improve access and quality
Sumarsono et al, 2023 [59]	mHealth Infrastructure	Develop NCD^e^ management systems
Nguyen, 2023 [60]	Health management information	Use DHIS2^f^ for health care
Thomas et al, 2023 [61]	mHealth qualitative research	Optimize mHealth solutions
De and Pradhan, 2023 [62]	Maternal and neonatal health care	Evaluate mobile tech impact
Alfian et al, 2023 [63]	Pharmaceutical care	Enhance health care access

^a^HIS: health information systems.

^b^mHealth: mobile health.

^c^HRH: human resource for health.

^d^HMIS: health management information systems.

^e^NCD: noncommunicable disease.

^f^DHIS2: Digital Health Information Systems 2.

Contributions related to health care facilities were primarily aimed at improving access to health care services, with 2 (5.88%) of the 34 papers [43,48] focusing on this topic. In contrast, communication networks were prominently featured across various interventions, including mHealth monitoring, educational platforms, infrastructure solutions, and digital health innovations. These topics were represented in 9 papers [30,36,37,44,45,49,61-63], or 26.47% of the total. Health technologies, encompassing areas such as eHealth, enterprise architecture, and mHealth solutions [39,47], were the focus of 6 (17.65%) papers [39,42,47,53-55].

Information systems emerged as the most frequently discussed topic, with initiatives such as health information systems and the management and utilization of platforms like District Health Information System 2 ([31,38,40] being covered in 12 (35.29%) papers [32,33,35,38,40,41,50-52,56,58,60]. In terms of quality assurance mechanisms, which include discussions on data quality, 3 (8.82%) papers [32,50,52] addressed this issue. Governance and legislation, in the context of digital health transformation, were represented by only 2 (5.88%) papers [57,59], indicating a limited focus on regulatory frameworks in this corpus.

#### Nomenclatures in Papers Contributing to UHC

The titles of the articles in this study were analyzed using both quantitative and qualitative approaches. A simulated generation of a word cloud (see Multimedia Appendix 1) highlighted frequently used terms such as “health,” “developing,” “information,” “countries,” and “systems,” indicating a focus on digital health interventions in resource-limited settings. Table 3 shows the most frequently terms (more than 10 times) in quantitatively order. The two words—“health” (36 mentions) and “developing” (26 mentions)—appear more frequently in the literature. Qualitatively, these terms underscore the growing importance of mobile technologies and digital systems in enhancing health care delivery and supporting UHC, particularly LMICs where access to health care remains a challenge. This combination of analysis reveals both the central themes in the field and their practical implications in advancing digital health solutions.

**Table 3:** Most frequent key terms identified in the selected studies.

Words	Frequency, n
Health	36
Developing	26
Information	24
Countries	23
System	22
Mobile	11

**Figure 2:**
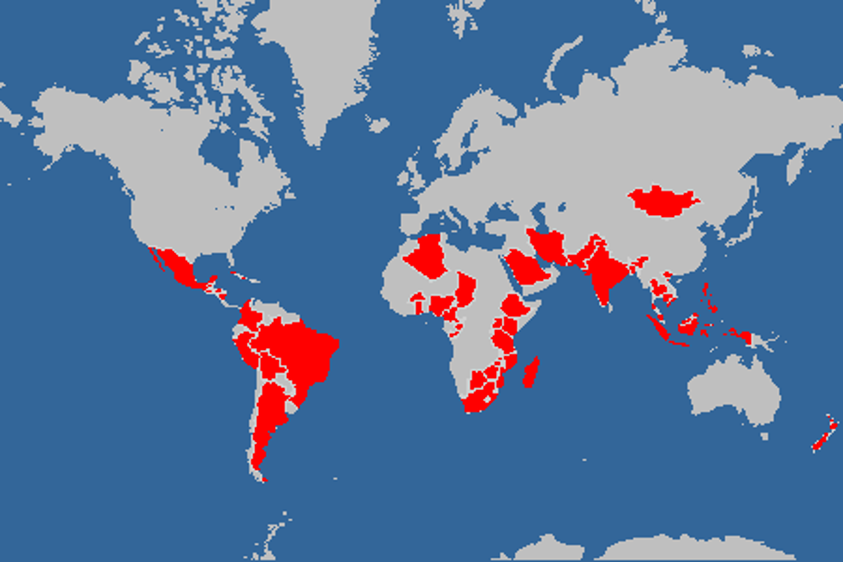
Countries where the studies were conducted.

The countries where the various studies were carried out are shown in Figure 2. There were 46 countries in total, distributed as follows: 20 in Africa, 12 in Asia, 11 in Latin America, and 3 in Oceania.

## Discussion

### Solution of Digital Health Initiatives in LMICs: Early Developments (1990s-2000s)

During the early years of digital health initiatives in LMICs, efforts were primarily focused on establishing sustainable health information systems [30,31]. Organizations such as the Health Information Systems Program (HISP) played a significant role in driving these initiatives, aiming to design and support the implementation of robust health information systems in low- and middle-income countries [32,34]. The HISP, initiated by the Department of Informatics at the University of Oslo in the mid-1990s, focused on improving data collection, management, and communication between health care institutions [35].

### Introduction of Basic Digital Health Technologies

The integration of fundamental digital health technologies marked a significant advancement in health care accessibility, especially in remote regions with limited traditional services. For instance, initiatives like text message–based platforms for health education in rural areas exemplify this transformative trend [44]. These platforms use the widespread availability of mobile phones to disseminate crucial health information, effectively bridging the gap in health care access and empowering individuals with knowledge for improved health outcomes.

In a qualitative study investigating the feasibility of implementing a mobile text message reminder system for child vaccination in northwest Ethiopia, challenges such as limited mobile phone ownership, access to networks, electricity, and illiteracy among the target population were identified [40]. However, stakeholder collaboration, user orientation or training, and the client’s willingness to pay were recognized as potential facilitators [40]. This research underscores the importance of considering these factors when introducing mHealth interventions in resource-limited settings.

### Focus on Addressing Specific Health Challenges

A targeted approach to addressing specific health challenges emerged in the rollout of SMS-based platforms for malaria diagnosis and treatment in sub-Saharan Africa [45]. This focused strategy harnesses mobile technology to enable prompt detection and intervention, significantly contributing to malaria control efforts [45]. Such initiatives highlight the potential of digital solutions to address pressing health issues and enhance health care delivery in underserved communities.

#### Transition Period (2010s)

The transition period of the 2010s witnessed significant growth in the field of digital health, driven by increased mobile phone penetration and the development of interactive mHealth apps. Studies during this time focused on scalability, sustainability, and the integration of mobile technology into health care systems [51]. Efforts were directed towards improving health information management, enhancing data collection and dissemination, and supporting health care delivery at various levels [38,39]. This period marked a shift toward more comprehensive strategies aimed at strengthening health information systems and supporting UHC objectives.

#### Recent Advancements (2020s)

In the recent advancements of the 2020s, digital health interventions continued to evolve with a focus on factors influencing sustainability, readiness for digital health transformation, and the roles of digital health interventions in strengthening health systems [39,55]. Studies explored the effectiveness of mobile technology in maternal and neonatal health care, the development of mHealth infrastructure for noncommunicable diseases, and the use of digital health interventions to improve immunization data collection and transmission [59,61]. Efforts were also made to identify factors influencing the successful integration of digital health within local health systems and to assess the impact of digital health interventions on health outcomes in low-resource settings [62,63]. These advancements reflect ongoing efforts to leverage technology to enhance health care delivery and support UHC goals in the digital age.

#### Rapid Deployment of Telemedicine Services to Treat COVID-19 Cases in Latin America

The COVID-19 pandemic has put unprecedented pressure on Latin American health care systems, requiring rapid and innovative responses to ensure adequate medical care while mitigating the risk of virus transmission. One of these responses has been the rapid rollout of telemedicine services to treat COVID-19 cases across the region [64].

Telemedicine, where health care services are delivered remotely via digital communication technologies, has proven to be an important tool for managing the increase in COVID-19 patients, alleviating the burden on health care facilities, and maintaining continuity of care amid lockdowns and social distancing measures. This rapid rollout of telemedicine services has been particularly important in Latin America, where traditional health care systems have faced unprecedented challenges due to the pandemic. Countries across Latin America have used telemedicine to enable remote consultations, triage COVID-19 cases, monitor patient health remotely, and provide medical advice and prescriptions without the need to visit health care facilities in person. Using telemedicine platforms, health care professionals can assess COVID-19 symptoms, provide guidance on self-isolation and symptom management, and ensure timely medical intervention for severe cases while reducing the risk of virus transmission in crowded health care facilities.

The use of telemedicine services in Latin America during the COVID-19 pandemic has demonstrated several important benefits. First, it has expanded access to health care services, especially for vulnerable populations in remote or underserved areas with limited access to traditional health care facilities. For example, in Bolivia, telemedicine services were introduced in the public sector to educate, assess, and analyze COVID-19 cases, resulting in approximately 200,000 teleconsultations in the first 100 days of implementation. Second, telemedicine has helped to relieve overburdened health care systems by reducing the number of nonurgent visits to hospitals and clinics, thus conserving resources for critical care [64]. Third, telemedicine has enabled timely intervention and monitoring of patients with COVID-19, resulting in better health outcomes and lower mortality rates. However, the rapid adoption of telemedicine services in Latin America also faces challenges and limitations. Unequal access to digital technologies and internet connectivity, particularly among low-income and rural populations, has prevented widespread adoption. In addition, concerns about privacy, security, and the legal framework for telemedicine practices have emerged as critical issues that need to be addressed to ensure the ethical and effective delivery of telemedicine services in the region [65].

The rapid adoption of telemedicine services to treat COVID-19 cases in Latin America has been instrumental in expanding access to health care, reducing the burden on health care systems, and improving patient outcomes during the pandemic. To fully realize the potential of telemedicine in promoting equitable and effective health care across the region, further efforts to address existing challenges and strengthen the regulatory framework are essential.

#### Accelerating Adoption and Innovation: Integration of Digital Contact Tracing Apps in Southeast Asia

The rapid deployment of digital contact tracing apps represents a pivotal innovation in Southeast Asia’s efforts to combat infectious diseases like COVID-19. For instance, countries in the region have swiftly embraced the use of these apps to monitor and manage the spread of the virus, mirroring global trends in technological response to the pandemic. One notable example is the integration of digital contact tracing apps in countries like Thailand and Myanmar [66]. Despite initial reluctance among citizens due to privacy concerns and government surveillance, these apps have become essential tools for tracking and containing outbreaks. However, challenges persist in ensuring widespread adoption and accurate usage.

In Thailand, recent protests against government policies have underscored the delicate balance between public health imperatives and individual rights. Similarly, Myanmar’s military coup and subsequent internet suppression have raised questions about the role of technology in governance and civil liberties [66].

The integration of digital contact tracing apps in Southeast Asia exemplifies the region’s commitment to innovation in disease control. However, it also highlights the complex interplay between technology, governance, and societal trust, underscoring the need for transparent and accountable approaches to public health interventions.

#### Key Milestones and Successes Contributions to UHC

Numerous successful digital health interventions identified in this study have contributed to advancing UHC globally, particularly in areas such as health service delivery systems, health workforce, facilities, communication networks, health technologies, information systems, quality assurance mechanisms, and governance and legislation [29]. These interventions have addressed the crucial need for improved access to health care and have shown promising outcomes. Azubuike and Ehiri [30] pioneered the use of digital platforms in health care, laying the foundation for subsequent innovation and wider adoption of digital health interventions. Similarly, Braa et al [31] and Kimaro and Nhampossa [32] conducted pioneering studies demonstrating the effectiveness of digital solutions in improving health care access and delivery, particularly in resource-constrained settings. Their work emphasizes the role of information and communication technology in overcoming barriers to health care and extending services to underserved populations.

Simba and Mwangu [33] emphasized the importance of flexible standards in implementing digital health solutions that lead to scalable and adaptable systems that meet diverse health care needs. Building on these foundations, Weibel et al [36] investigated innovative approaches such as biometric fingerprint identification systems to improve patient identification and health care delivery in challenging environments such as Chad. Asangansi and Braa [38] demonstrated the effectiveness of open-source mobile platforms integrated into existing health care infrastructure, highlighting the potential of mobile technology in bridging access gaps and improving service delivery in countries such as Nigeria. In addition, researchers such as Blaya et al [39] emphasized the critical role of information technology in transforming health care delivery models and optimizing patient care and health care processes.

Initiatives such as those by Nguyen and Nyella [40] in Tanzania and Vietnam are examples of the successful implementation of District Health Information Systems and HISPs that significantly improve data management and decision-making processes, thereby advancing health care goals. In addition, O’Connor et al [45] in Malawi show that the introduction of mHealth apps and technologies has expanded access to health services and empowered patients to participate in their health management actively.

Researchers such as Gebre-Mariam and Fruijtier [47] have emphasized the importance of enterprise architecture and IT governance in optimizing health systems, particularly in resource-constrained settings such as Ethiopia. Taken together, these case studies and initiatives represent important milestones on the road to UHC. They illustrate the transformative impact of digital health interventions in improving access to health care, improving service delivery, and ultimately realizing the goal of UHC for all.

#### Challenges and Barriers

The realization of UHC in LMICs, particularly in Africa, faces numerous challenges. These hurdles include a wide range of problems, from inadequate health infrastructure and governance deficiencies to a shortage of qualified health professionals [67,68]. In addition, rapid urbanization, limited resources, and the persistence of diseases such as HIV/AIDS and malaria further complicate the quest for equitable health care. This is compounded by glaring disparities in access to health care due to the urban-rural divide and the socioeconomic gap, which exacerbate the difficulties. Given this multifaceted landscape, it is imperative to explore the barriers that impede progress toward UHC and address the challenges head on. In this way, we can develop strategies that promote inclusive and sustainable health systems tailored to the needs of these regions.

#### Challenges in Realizing UHC in LMICs: Case of Africa

Regions in Africa face significant hurdles in building sustainable and equitable health systems. The continent’s inadequate health infrastructure and institutions hinder a rapid response to emerging health problems. Factors such as high turnover in senior health authorities, inadequate resources, poor governance, and inefficient implementation further hinder progress [67,68]. Although the private sector makes a significant contribution to health care, there is a notable gap in communication and information sharing between the commercial and public sectors. Outdated legal frameworks and limited resources exacerbate enforcement problems [69,70]. Private health facilities often disregard inspections and have no standardized criteria for medical training. Rapid urbanization exacerbates the strain on health care systems and leads to increased demand for urban hospitals. There is also a severe shortage of qualified health care staff in Africa, which further complicates health care provision. The challenges of reducing child mortality and combating diseases such as HIV/AIDS and malaria persist and are exacerbated by inefficient resource allocation, governance problems, and a lack of evidence-based strategies [68,71]. Inequalities in access to health care persist, particularly in terms of the urban-rural divide and socio-economic disparities, further hindering progress in health equity [72,73].

#### Bridging Health Inequalities: Role of Digital Health

Digital technologies have found widespread application in public health over the past two decades, offering opportunities to improve the speed and cost-effectiveness of health care services [74]. Smart wearables, social media, mobile apps, big data, and AI are among the digital tools being used to transform public health services [75]. International and regional health authorities have developed policy frameworks to capitalize on the potential benefits of digital technologies to improve health outcomes. Digital health innovations are particularly relevant for neglected and socio-economically disadvantaged populations as they improve access to medical services, health information, and overall health awareness. Digital solutions facilitate access to patient records and improve health care decision-making and the effectiveness of therapies. New technologies such as genomics, AI, and remote patient monitoring hold great promise for improving diagnosis, therapies, and precision medicine models. Telemedicine and remote patient monitoring have significantly contributed to access to health care for underserved populations, particularly in maternal care and disease management [76,77]. In addition, the digital transformation of health care is transforming traditional medical services, pharmaceutical research, and health care systems, leading to improved efficiency and effectiveness.

### Recommendations

To remove barriers to the uptake of digital health care, targeted health care policies that reflect sociocultural norms and practices need to be implemented. Measures such as affordable internet access, infrastructure development, and digital literacy programs are essential to facilitate access to digital health tools, especially for economically disadvantaged populations [74]. Investment in human resource development, capacity building, and better remuneration of health workers is critical to improving access to health care in Africa. Monitoring and evaluation systems must be prioritized to address implementation challenges and improve health care delivery [78]. In addition, technology companies and development organizations should invest in the research and development of assistive technologies to bridge the digital divide and ensure equitable access to health care.

### Future Directions and Opportunities

In terms of future directions and opportunities for digital health in LMICs, emerging technologies such as AI are very promising. For example, the integration of AI algorithms for early detection of noncommunicable diseases in Southeast Asia could significantly improve proactive interventions, thereby improving health outcomes and reducing health care costs. International collaborations and partnerships play a critical role in leveraging expertise and resources to address health care challenges. Joint initiatives between governments and technology companies to develop scalable digital health solutions in Africa can improve access to and quality of health care and align with the goals of UHC.

Engaging and empowering the community is critical to the successful implementation of digital health. By empowering local health workers to train communities in the use of digital health tools for disease prevention in the Caribbean, ownership, trust, and sustainability can be fostered, leading to greater uptake and effectiveness. Strategies that ensure equitable access and inclusion of the population are of paramount importance. Implementing subsidized smartphone distribution programs in South America can reduce barriers to technology use in low-income populations, narrow the digital divide, and promote health equity. Capitalizing on these future directions and opportunities can pave the way for transformative advances in health care delivery and outcomes in LMICs.

### Limitations

This study reviews digital health interventions targeting UHC in LMICs, identifying 30 studies from 1999 to 2024 using the Research Rabbit AI Mapping Tool for papers and following PRISMA guidelines. These studies highlight the contributions of digital health interventions to UHC, emphasizing improvements in health data management, access to health care, and system sustainability across diverse geographical regions and technological domains.

However, the study is subject to certain limitations. While comprehensive, the scope may have inadvertently excluded relevant studies due to search strategies and indexing limitations. For instance, relatively few papers were identified from curated *JMIR Publications* sections, such as “mHealth in the Developing World and LMICs, Underserved Communities, and for Global Health” (*JMIR mHealth and uHealth*); “Clinical Informatics in Low-Resource Settings and the Developing World” (*JMIR Medical Informatics*); and “Health Services in Resource-Poor Settings and LMICs” (*JMIR Public Health and Surveillance*). This gap may be attributed to indexing challenges or the specificity of search terms. In addition, the study is susceptible to language, potential publication, and time biases, as only studies published in English and within specific timeframes were included.

Despite these limitations, the study provides valuable insights into the evolving landscape of digital health interventions in LMICs and their potential to advance UHC goals. Addressing these gaps in future research could enhance the comprehensiveness and impact of studies in this critical field, contributing to more equitable and effective health strategies globally.

### Conclusions

To conclude, digital health initiatives in LMICs have spanned decades of innovation, adaptation, and perseverance. From early developments in establishing sustainable health information systems to recent advances in the use of cutting-edge technologies, this reflects a collective commitment to improving accessibility, equity, and health outcomes.

The development of digital health interventions underscores their transformative potential in bridging long-standing gaps in health care delivery. Initiatives such as SMS-based health education platforms, remote patient monitoring, and digital contact tracing apps have shown tangible benefits in improving health awareness, disease management, and controlling disease outbreaks. These achievements have not only enhanced individual health outcomes but have also contributed to broader public health goals, including the realization of UHC.

Amid the progress, however, are daunting challenges requiring concerted action and innovative solutions. Inadequate infrastructure, scarce resources, governance issues, and inequitable access to health care remain barriers to equitable health systems in LMICs. The rapid adoption of digital health technologies faces hurdles such as unequal access to technology, privacy concerns, and regulatory complexity that require targeted action and policy frameworks to address effectively.

To overcome these challenges and realize the full potential of digital health, a multifaceted approach is essential. This includes prioritizing investment in infrastructure development, digital literacy programs, and human capacity building. It also includes fostering collaboration between governments, technology companies, health care providers, and local communities to ensure inclusive and sustainable solutions. In addition, proactive measures on privacy, security, and ethical considerations are essential to build trust in digital health interventions. The future of digital health in LMICs is bright. Emerging technologies such as AI, telemedicine, and big data analytics offer unprecedented opportunities to revolutionize health care, disease prevention, and health promotion. By harnessing these innovations responsibly and equitably, it is possible to accelerate progress towards universal health care and improve health outcomes for all.

The evolution of digital health in LMICs is a testament to human ingenuity, resilience, and collective determination to overcome challenges and create a healthier and more equitable world.

## Appendix

Multimedia Appendix 1 Worcloud.

Multimedia Appendix 2 PRISMA Checklist.

## Abbreviations

AI: artificial intelligence
EHR: electronic health record
HISP: Health Information System Program
LMIC: Low- and Middle-Income Country
mHealth: mobile health
PICO: Population, Intervention, Comparison, and Outcome
PRISMA: Preferred Reporting Items for Systematic Reviews and Meta-Analyses
UHC: universal health coverage
