# Supplementary figures and images for: 25 Years of Digital Health Toward Universal Health Coverage in Low- and Middle-Income Countries: Rapid Systematic Review
Source: J Med Internet Res. 2025 May 29;27:e59042. doi: 10.2196/59042 (PMC12163355; doi:10.2196/59042)

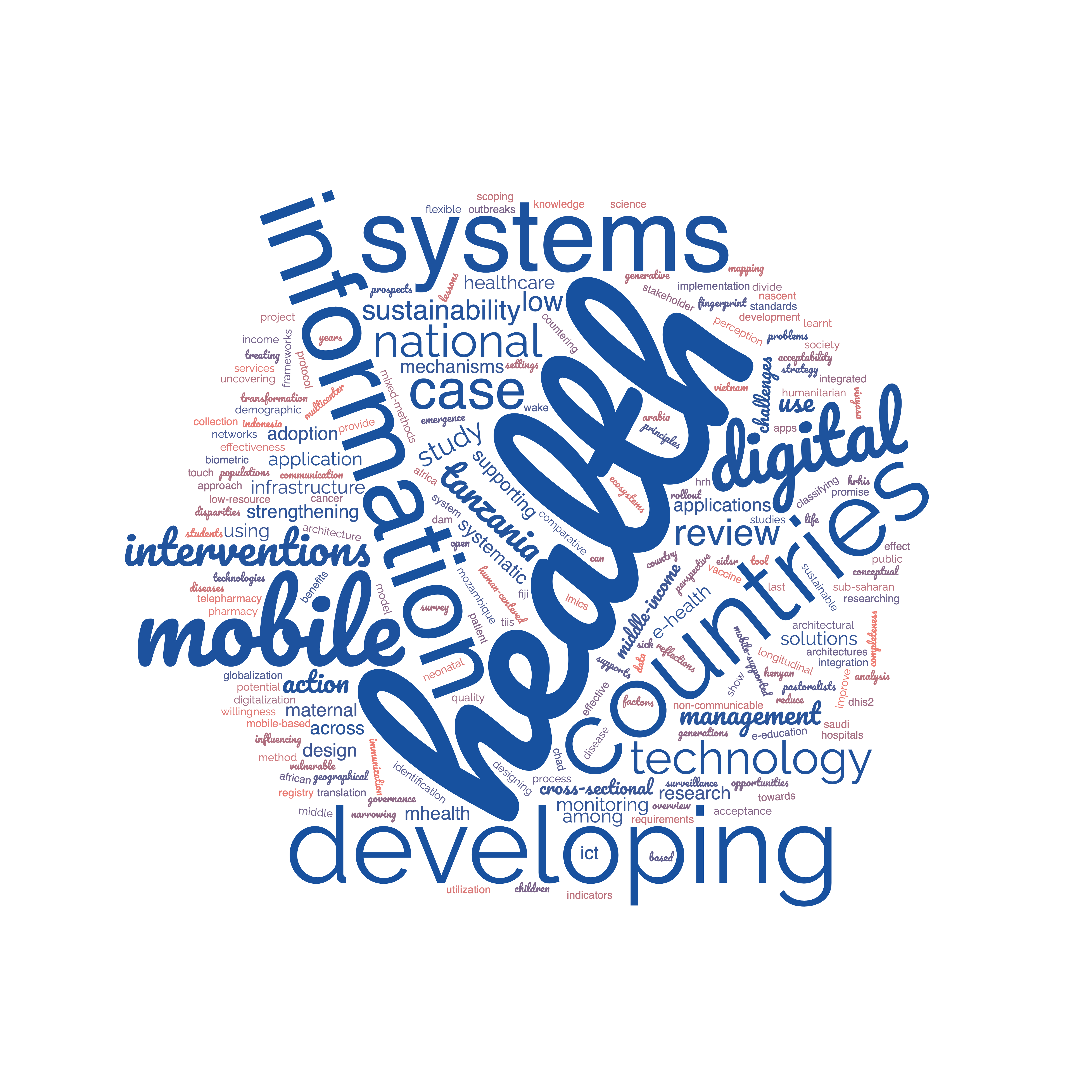

Supplement: Multimedia Appendix 1 [file jmir_v27i1e59042_app1.png]
